# Systematic Review and Meta-Analysis of the Efficacy and Safety of Enhanced Recovery After Surgery vs. Conventional Recovery After Surgery on Perioperative Outcomes of Radical Cystectomy

**DOI:** 10.3389/fonc.2020.541390

**Published:** 2020-09-23

**Authors:** Dongxu Zhang, Kai Sun, Tianqi Wang, Gang Wu, Jipeng Wang, Yuanshan Cui, Jitao Wu

**Affiliations:** ^1^Department of Urology, Yantai Yuhuangding Hospital, Qingdao University, Yantai, China; ^2^Department of Urology, Beijing Tiantan Hospital, Capital Medical University, Beijing, China

**Keywords:** meta-analysis, radical cystectomy, ERAS (enhanced recovery after surgery), CRAs, RCT—randomized controlled trial

## Abstract

**Background and objective:** Radical cystectomy has been characterized as the most difficult operation in urology because of the complex surgical procedures and postoperative complications. Enhanced recovery after surgery (ERAS), which reduces the incidence of perioperative complications, has been widely used in clinical surgery. Herein, we performed a meta-analysis to evaluate the efficacy and safety of ERAS vs. conventional recovery after surgery (CRAS) on perioperative outcomes of radical cystectomy.

**Methods:** We performed a systematic search of randomized controlled trials (RCTs) in the following databases: Medline, Embase, and the Cochrane Controlled Trials Register, based on the PICOS strategy. The reference lists of the retrieved studies were further surveyed for relevant publications.

**Results:** Our search yielded seven RCTs containing 813 patients. The ERAS group was found to have better performance in the following parameters: length of hospital stay [mean difference (MD) = −1.12, 95% confidence interval (CI): −1.80 to −0.45, *P* = 0.001], time to first flatus (MD = −0.70, 95% CI: −0.98 to 0.41, *P* < 0.00001), and time to regular diet (MD = −0.12, 95% CI: −1.76 to −0.28, *P* = 0.007). However, there were no significant differences between the two groups in major complications [odds ratio (OR) = 0.91, 95% CI: 0.63 to 1.34, *P* = 0.64], readmission (OR = 1.15, 95% CI: 0.65 to 2.01, *P* = 0.63), ileus (OR = 0.75, 95% CI: 0.44 to 1.28, *P* = 0.29), wound infection (OR = 0.56, 95% CI: 0.31 to 1.01, *P* = 0.05), mortality (OR = 0.69, 95% CI: 0.24 to 1.99, *P* = 0.49), or time to first bowel movement (MD = −0.55, 95% CI: −1.62 to 0.53, *P* = 0.32).

**Conclusion:** ERAS reduced the length of hospital stay, time to first flatus, and time to regular diet after cystectomy. Compared to CRAS protocols, ERAS protocols do not increase the risk of adverse events.

## Introduction

Although the conventional recovery after surgery (CRAS) protocol on perioperative outcomes of radical cystectomy includes preoperative prolonged fasting and preoperative mechanical bowel preparation, the effect of this protocol is not apparent. Major operations are still associated with undesirable sequelae, such as cardiovascular complications, deep venous thrombosis, wound infection, and prolonged convalescence. Enhanced recovery after surgery (ERAS) was first proposed by a Danish surgeon, Professor Henrik Kehlet (1, 2), and has been widely applied in postoperative recovery, to reduce perioperative stress and postoperative morbidity and shorten hospital stays (3). In recent years, the number of studies employing ERAS for radical cystectomy has rapidly accumulated.

Herein, we conducted a systematic review and meta-analysis of the existing literature to evaluate the safety and efficacy of ERAS vs. CRAS protocols on perioperative outcomes of radical cystectomy. We compared the changes in the length of hospital stay, time to first flatus, time to regular diet, complications, and other indices between the two protocols. We aimed to produce better planning and optimization of perioperative management.

## Materials and Methods

### Search Strategy

A systematic literature search of Medline, Embase, and Cochrane Controlled Trials Register databases was performed based on the PICOS strategy to collect relevant randomized controlled trials (RCTs) that applied ERAS and CRAS approaches after radical cystectomy. This systematic review and meta-analysis were strictly guided by the Preferred Reporting Items for Systematic Reviews and Meta-Analyses (PRISMA) checklist. This systematic review has been registered on PROSPERO (CRD42020162400). The following search terms were used: “ERAS,” “radical cystectomy,” and “RCTs.” We further scanned the reference lists of the included studies for additional candidate articles. The search strategy according to the focused PICOS question is presented in Table 1.

**Table 1:** Search strategy according to populations, interventions, comparators, outcomes, and study designs (PICOS).

	**Population**	**Intervention**	**Comparator**	**Outcomes**	**Study Design**
Inclusion Criteria	Patients underwent radical cystectomy.	Enhanced Recovery After Surgery (ERAS) protocol	Conventional recovery after surgery (CRAS) protocol	Length of hospital stay. Time to first flatus. Time to first bowel movement. Time to regular diet. Complications, Readmission, Ileus, Wound infection, Mortality.	Randomized Controlled Trials
Exclusion Criteria	Patients underwent transurethral resection of the bladder tumor. Patients with other concomitant malignancies. Patients with mental or cognitive disorders. Patients with a history of radiotherapy. Patients with gastrointestinal disease affecting feeding.	Not performed	Not performed	Leakage of urine. Deep venous thrombosis. Diarrhea. Hydronephrosis. Cardiovascular disease. Lung emboli.	Letters, comments, reviews, qualitative studies

### Inclusion Criteria

**Figure 1:**
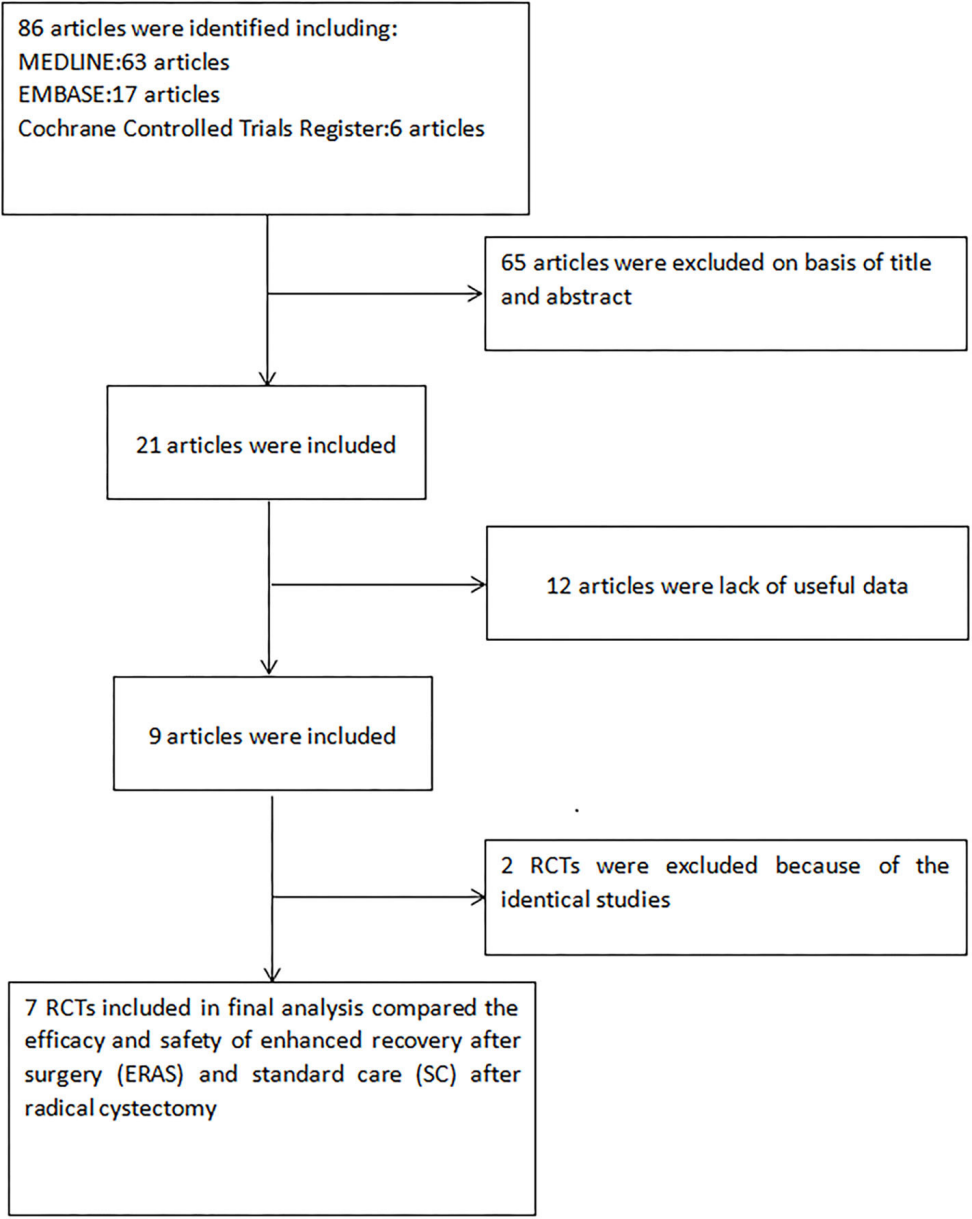
Flowchart of the study selection process. RCT, randomized controlled trials; ERAS, enhanced recovery after surgery; CRAS, conventional recovery after surgery.

Two authors independently completed the screening process for eligibility of candidate articles, and any differences were unified through discussion and a consultation with a third investigator. Eligible studies were identified according to the following inclusion criteria: (1) both the ERAS and CRAS protocols were analyzed on perioperative outcomes of radical cystectomy; (2) the full text of the article was available; (3) sufficient data were provided, including the number of participants and the number of each indicator. The inclusion criteria of the population of RCTs are stricter and of higher quality than other prospective and retrospective studies. If a study was analyzed in multiple publications, the latest was included in our meta-analysis. Figure 1 presents the flowchart for the selection process.

### Quality Assessment

The methodological quality of selected RCTs was evaluated using the Jadad scale. The quality of each study was evaluated based on the method of patient allocation, concealment of allocation, blinding method, and number of lost follow-up. Based on the guidelines published in the Cochrane Handbook for Systematic Reviews of Interventions V.5.1.0, the quality of each study was graded as follows: quality degree “A,” the study met all quality criteria and had a low-risk of bias; quality degree “B,” the study met most quality criteria and had a moderate risk of bias; and quality degree “C,” the study met few quality criteria and had a high risk of bias. All authors participated in the evaluation process and reached a consensus on the results.

### Data Extraction

The following outcomes of interest were obtained: (A) published time; (B) name of the first author; (C) recovery therapy of patient; (D) operation type; (E) sample size of each group; (F) differences in the following indicators: complications, readmission, ileus, wound infection, mortality, length of hospital stay, time to first flatus, time to regular diet, and time to first bowel movement.

### Statistical Analyses and Meta-Analysis

Statistical analysis was performed using the Review Manager software (RevMan, version 5.3.0, Cochrane Collaboration) (4), which included the number of patients with complications, readmission, ileus, wound infection, mortality, the mean length of hospital stay, mean time to first flatus, mean time to regular diet, and mean time to first bowel movement. The outcomes were calculated as standardized mean difference (SMD) for continuous variables and odds ratio (OR) for dichotomous variables with 95% confidence intervals (95% CIs) (5). The fixed-effects model was used for studies that were considered homogeneous, with a *P* > 0.05. On the contrary, the random-effects model was selected. The *I*^2^ statistic was used to measure heterogeneity. A *P* < 0.05 was considered statistically significant.

## Results

### Characteristics of Eligible Studies

Electronic database search yielded a total of 86 articles. After screening all titles and abstracts, 65 articles were eliminated. Next, 12 of 21 remaining articles were removed due to a lack of enough data. Two more studies with identical data sets were also excluded. Finally, seven RCTs (6–12) evaluating the recovery effect of radical cystectomy between ERAS protocols and CRAS protocols were included in the analysis (Figure 1). The baseline characteristics of the studies are presented in Table 2.

**Table 2:** Study and patient characteristics.

**First author, year**	**Country**	**Study interval**	**Design**	**Recovery protocol**		**Matching/comparable**	**Inclusion population**
				**ERAS**	**CRAS**		
(11)	Canada	_	RCT	12	15	1,2,3,4,8	27 consecutive patients scheduled for RC for advanced BC were randomized prospectively
(6)	Germany	2010–2012	RCT	62	39	1,2,3,4,8	A total of 101 patients who underwent radical cystectomy for bladder cancer between 2010 and 2012 were enrolledin the study
(12)	China	2014–2016	RCT	144	145	1,2,3,4,5,8	Bladder cancer patients scheduled for curative treatment by RC were recruited from urology centers in the Chinese Bladder Cancer Consortium (CBCC) between October 2014 and July 2016
(8)	Denmark	2011–2013	RCT	50	57	1,2,3,4,7,8	All patients (*n* = 158) scheduled for RC owing to localized muscle-invasive bladder cancer or high-risk non-muscle-invasive bladder cancer from May 2011 to February 2013 at Aarhus University Hospital in Denmark were enrolled
(10)	China	2006–2009	RCT	47	39	1,2,5,8	86 patients receiving ileal urinary diversion in center from March 2006 to January 2009
(9)	USA	2011–2014	RCT	50	52	1,2,4,6,7,8	All adult patients undergoing radical cystectomy and urinary diversion for bladder cancer at two institutions were invited to participate in this randomized trial
(7)	Germany	2010–2012	RCT	62	39	1,2,6,7,8,9	101 patients who underwent radical cystectomy for bladder cancer between 2010 and 2012 were enrolled in the study

*ERAS, enhanced recovery after surgery; CRAS, conventional recovery after surgery; RC, radical cystectomy; 1, age; 2, sex; 3, body mass index; 4, American Society of Anesthesiologists score; 5, history of previous surgery; 6, neoadjuvant chemotherapy; 7, clinical stage; 8, operation type; 9, number of lymph nodes removed*.

### Quality of Eligible Studies

The seven studies were all RCTs and had an appropriate calculation of the sample size to analyze. The quality grade of each of the included study was A. Only one study explained the blinding method. In six of seven RCTs, the patient follow-up process was completed. The summary of the quality of the included studies is shown in Table 3.

**Table 3:** Quality assessment of individual study.

**Study**	**Allocation sequence generation**	**Allocation concealment**	**Blinding**	**Loss to follow-up**	**Calculation of sample size**	**Statistical analysis**	**Level of quality**	**ITT analysis**
(11)	A	A	B	0	Yes	ANCOVA	A	NO
(6)	A	A	B	0	Yes	ANCOVA	A	NO
(12)	A	A	B	0	Yes	ANCOVA	A	NO
(8)	A	A	A	0	Yes	ANCOVA	A	Yes
(9)	A	A	B	0	Yes	ANCOVA	A	NO
(10)	A	A	B	0	Yes	ANCOVA	A	NO
(7)	A	A	B	3	Yes	ANCOVA	A	NO

*A, all quality criteria met (adequate): low risk of bias; B, most quality criteria met (adequate): moderate risk of bias; ITT, intention to treat; ANCOVA, analysis of covariance*.

### Efficacy

#### Length of Hospital Stay

**Figure 2:**
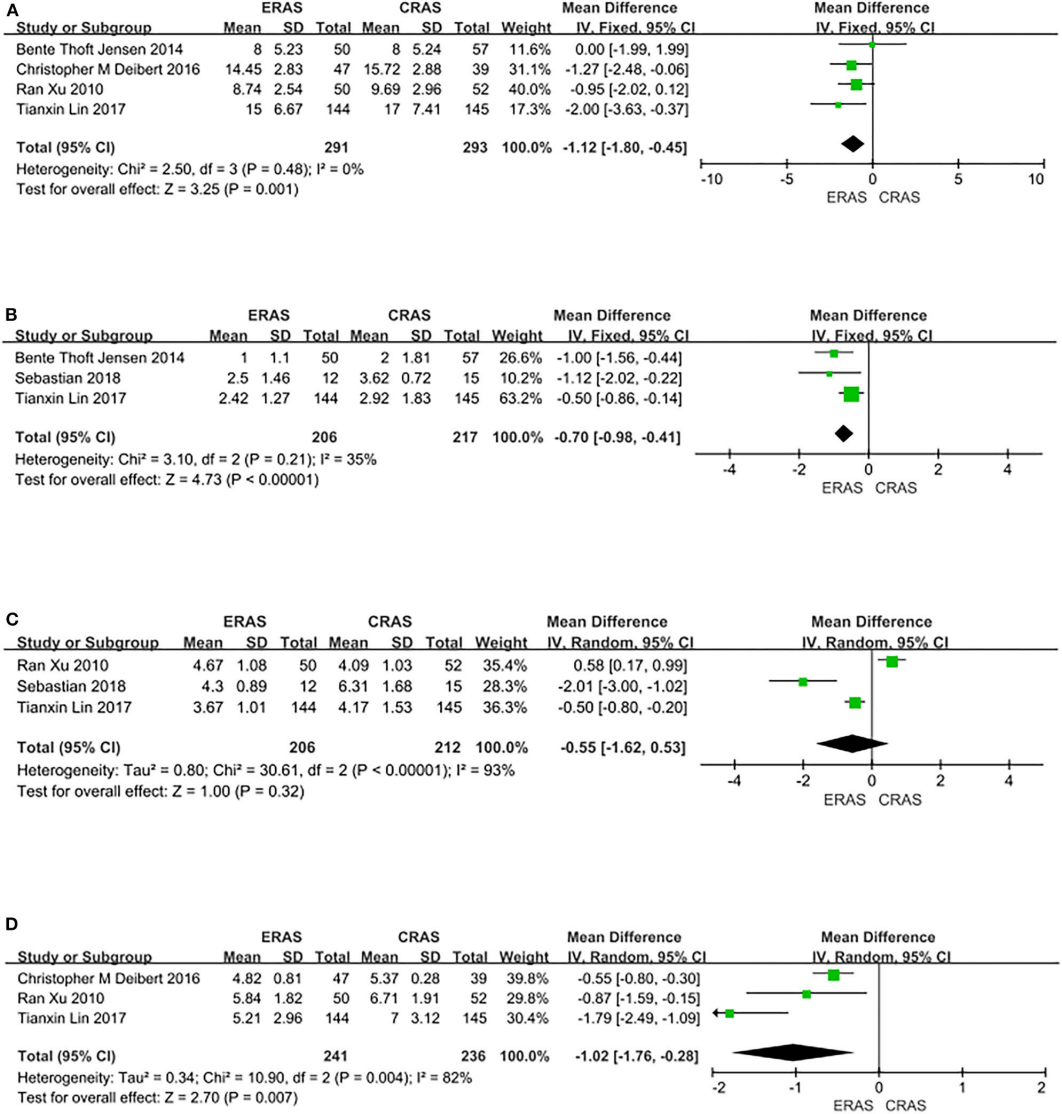
Forest plots showing changes in **(A)** length of hospital (days); **(B)** time to first flatus (days); **(C)** time to first bowel movement (days); and **(D)** time to regular diet (days).

Four RCTs covering 584 patients (291 in the ERAS group and 293 in the CRAS group) recorded the length of hospital stay. The pooled results from a fixed-effects model showed that the length of hospital stay was significantly reduced in the ERAS group compared with the CRAS group (MD = −1.12, 95% CI: −1.80 to −0.45, *P* = 0.001, Figure 2A).

#### Time to First Flatus

Three RCTs covering 423 patients (206 in the ERAS group and 217 in the CRAS group) reported the time to first flatus. The results revealed that the time to first flatus was greatly improved in the ERAS group than in the CRAS group (MD = −0.70, 95% CI: −0.98 to 0.41, *P* < 0.00001, Figure 2B).

#### Time to First Bowel Movement

Three RCTs covering 418 patients (206 in the ERAS group and 212 in the CRAS group) analyzed reported time to first bowel movement. The results from a random-effects model showed that there was no difference between the ERAS group and the CRAS group in the time to first bowel movement (MD = −0.55; 95% CI: −1.62 to 0.53, *P* = 0.32, Figure 2C).

#### Time to Regular Diet

Three RCTs covering 477 patients (241 in the ERAS group and 236 in the CRAS group) presented the time to regular diet. The findings from a random-effects model favored the ERAS group in the time to regular diet (MD = −0.12, 95% CI: −1.76 to −0.28, *P* = 0.007, Figure 2D).

### Safety

#### Complications

**Figure 3:**
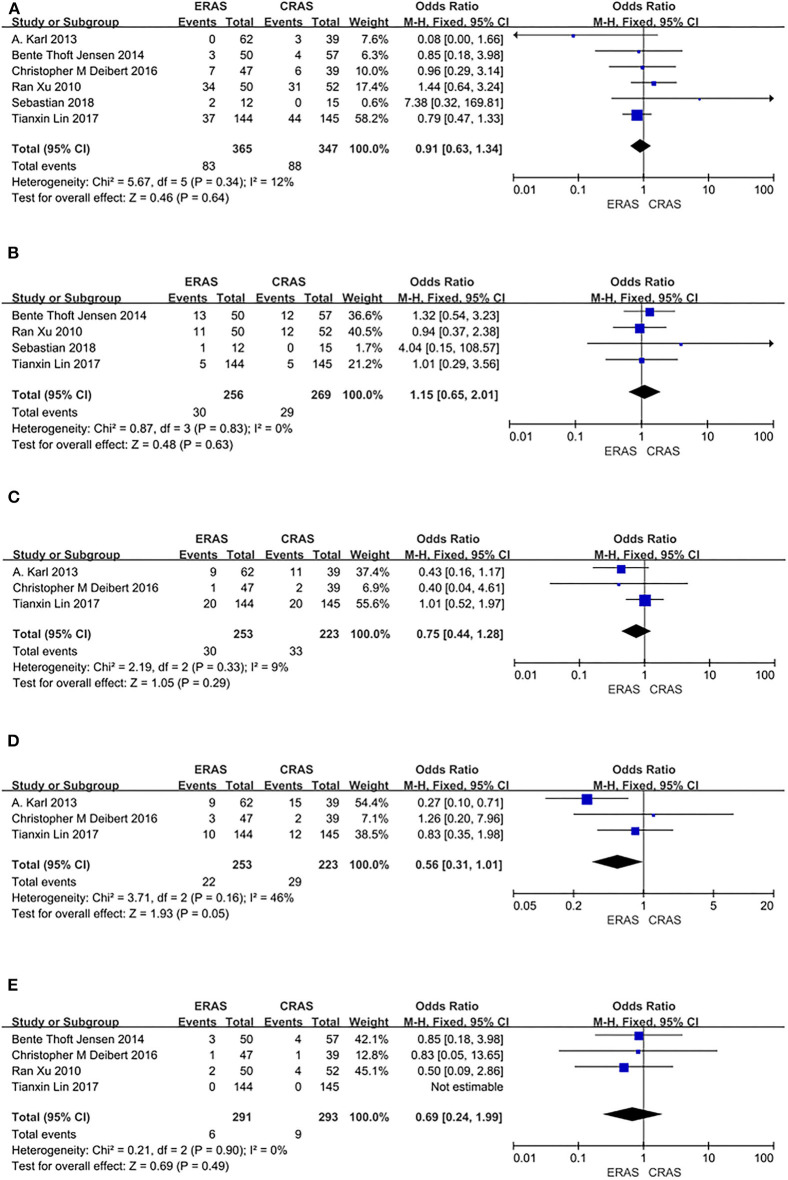
Forest plots showing changes in **(A)** complications; **(B)** type of complications: readmission; **(C)** type of complications: ileus; **(D)** type of complications: wound infection; and **(E)** mortality.

Six RCTs including 712 patients (365 in the ERAS group and 347 in the CRAS group) recorded postoperative complications. The fixed-effects model showed that the ERAS and the CRAS groups were similar in terms of the postoperative complications (OR = 0.63, 95% CI: 0.63 to 1.34, *P* = 0.64, Figure 3A).

#### Readmission

Four RCTs including 525 patients (256 in the ERAS group and 269 in the CRAS group) reported on readmission. The fixed-effects model showed that there was no significant difference between the ERAS group and the CRAS group in readmission (OR = 1.15, 95% CI: 0.65 to 2.01, *P* = 0.63, Figure 3B).

#### Ileus

Three RCTs including 486 patients (253 in the ERAS group and 233 in the CRAS group) reported on postoperative ileus. A fixed-effects model revealed that there was no difference between the ERAS group and the CRAS group in postoperative ileus (OR = 0.75, 95% CI: 0.44–1.28, *P* = 0.29, Figure 3C).

#### Wound Infection

Three RCTs including 486 patients (253 in the ERAS group and 233 in the CRAS group) reported data on wound infection. A fixed-effects model showed no significant differences between the ERAS group and the CRAS group in wound infection (OR = 0.56, 95% CI: 0.31 to 1.01, *P* = 0.05, Figure 3D).

#### Mortality

Three RCTs including 584 patients (291 in the ERAS group and 293 in the CRAS group) reported on mortality. A fixed-effects model analysis showed no significant difference in mortality between the two groups (OR = 0.69, 95% CI: 0.24 to 1.99, *P* = 0.49, Figure 3E).

## Discussion

Although the surgical approach to radical cystectomy has been optimized, it is still a challenging procedure and results in a long hospital stay and difficult postoperative recovery. At present, there is controversy concerning perioperative protocols in radical cystectomy. Besides, CRAS protocols are still preferred by most surgeons. Although ERAS has been reported in the literature to accelerate postoperative recovery of patients such as reducing the length of stay and complication rates (13), its application in the perioperative period is still limited (14). Some studies have confirmed that several measures of the CRAS protocols are unnecessary. For instance, traditional bowel preparation is not essential; instead, it has been associated with greater morbidity (15, 16). Since the concept of ERAS was introduced in the 1990s, it has been gradually implemented globally by urologists. Compared with CRAS, ERAS protocols promote postoperative recovery via faster peristalsis, early resumption of oral intake, and reduction or stoppage of the application of nasogastric tube, to reduce the length of hospital and readmission (17). Patients applying ERAS procedures have been shown to exhibit faster return of bowel function, shorter length of hospital stay, and fewer complications (6, 18–21).

In recent years, ERAS has gained increasing attention in various surgical disciplines. Lohsiriwat et al. (22) concluded that the ERAS pathway was beneficial in the rehabilitation of patients undergoing emergency resection for obstructive colorectal cancer. Kleiman et al. (23) found that the ERAS pathway for elective cesarean delivery showed greater improvements in analgesic and recovery outcomes. Also, Sartori et al. (24) proved that ERAS pathways have several advantages for patients undergoing abdominal wall reconstruction. Recently, numerous studies have been employed to evaluate the impact of ERAS protocol on radical cystectomy. For instance, Giannarini et al. (25) found that implementation of the ERAS protocol on radical cystectomy accelerated postoperative recovery and did not increase the risk of postoperative complications. Tyson et al. (20) showed that compared with the conventional protocol, the ERAS protocol had advantages in patients undergoing cystectomy and urinary diversion. Xiao et al. (26) demonstrated that implementation of the ERAS protocol could decrease the incidence of postoperative complications after radical cystectomy. However, the ERAS protocols were not suitable for every patient because of the diverse individual characteristics.

In the present study, the ERAS protocols shortened the length of hospital stay, time to first flatus, and time to regular diet. In two RCTs (6, 11), the ERAS group markedly benefited from a lower incidence of cardiovascular disease. The ERAS was also superior to CRAS in a lower incidence of pulmonary embolism (6, 10). Karl et al. (6) found that patients following the ERAS program had significant superiority in the prevention of deep venous thrombosis. However, the incidence of complications, readmission, ileus, wound infection, mortality, and the time to first bowel movement were not different between the ERAS and the CRAS patients. The pooled results in our meta-analysis demonstrated that the ERAS protocols significantly promoted efficacy in perioperative outcomes of radical cystectomy. ERAS protocols are suggested to enhance early mobilization and resumption of oral intake. All these measures can promote gastrointestinal peristalsis and regulate metabolism. Moreover, our results showed that the safety of perioperative outcomes in the ERAS group was similar to that of the CRAS group; thus, the ERAS protocols did not increase perioperative risk. Our results are of great importance for clinical and scientific practice.

ERAS is a standardized mechanism for fast recovery after surgery to reduce stress response and promote physiological recovery in patients (27). Based on the clinical experience and expertise, the essence of ERAS is multi-model, multidisciplinary collaboration. In terms of evidence-based medicine and perioperative care, ERAS fundamentally challenges the traditional approach in surgical care (28, 29). ERAS is not only a change in the surgical concept but also a change in the mode of surgical treatment. Specifically, ERAS deals with all phases of the perioperative period, including preoperative patient education, preoperative medical optimization, the omission of preoperative bowel preparation, early mobilization, and early oral diet (30–33). Previous studies have explained how the physiological mechanism of ERAS improves the perioperative effect. For instance, preoperative carbohydrate loading enhances perioperative insulin sensitivity and maintains lean body mass and muscle strength (34); the maintenance of visceral perfusion to reduce the incidence of ileum can be achieved through target-oriented fluid management (35, 36); body temperature monitoring, the maintenance of normothermia early mobilization, and early oral feeding reduce complications by maintaining body homeostasis (14). In addition, early ambulation, one of the core measures of ERAS, can ensure normal respiratory function and accelerate the flow of oxygen in the entire body. Many studies have also confirmed the strengths of ERAS in lowering intraoperative blood loss (37). It is necessary to apply the various measures of the ERAS protocols to the perioperative period of patients of radical cystectomy. The specific rehabilitation protocols should be tailored to each patient's specific circumstances. It is the combination of these approaches that greatly reduces the stress response, organ dysfunction, and the time required for a full recovery.

Furthermore, ERAS has a unique preponderance in other aspects. The ERAS protocols may increase the hospital bed turnover rate and allocate medical resources more scientifically and efficiently. The implementation of ERAS contributes to the improvement of the hospital's quality management system. ERAS protocols were associated with the improvement of the level of medical technology, standardization of the process of diagnosis and treatment, and reduction of the workload of medical staff.

Currently, there are some disputes about the ERAS protocols largely regarding the safety of early discharge. The outcomes of our meta-analysis revealed that the safety of the ERAS protocols was similar to that of the CRAS protocols. Nevertheless, to ensure the implementation of ERAS, follow-up after discharge is also of vital importance. ERAS is not only the tendency of the future perioperative period of surgery but also the key to innovation in the history of medical development. In the perioperative period of patients of radical cystectomy, the ERAS protocols should be widely used to promote recovery.

In all the RCTs included in our study, age, sex, and operation type of the patients were the indicator types analyzed. Concerning operation type, 538 patients from the seven RCTs (6–12) underwent open radical cystectomy. Three RCTs (8, 9, and 12) included 81 patients who underwent robot-assisted radical cystectomy. One RCT (12) had 215 patients who underwent laparoscopic radical cystectomy. Besides, urinary diversion in the included RCTs was mainly divided into three types: ileal conduit, neobladder, and continent reservoir, where 515 patients received ileal conduit, 244 patients received neobladder, and 3 patients received continent reservoir. The results of each study also emphasized on the different elements of ERAS. Five RCTs (6, 8, 9, 11, and 12) reported the American Society of Anesthesiologists (ASA) score as an indicator, and four RCTs (6, 8, 11, and 12) included body mass index (BMI) in their analysis.

Compared with the previous studies (20, 25, 26), our meta-analysis has the following advantages. First, our study involved seven RCTs. Our data were collected entirely from the RCTs, which have a low risk of bias. Second, the quality of RCTs included in our meta-analysis was very high. Finally, the results of the RCTs supported our study, suggesting that our findings could provide the basis for daily clinical practice. However, our study also has several shortcomings. First, because the RCTs included did not perform a subgroup analysis based on the operation type, we grouped all the patients implementing the ERAS protocols into one group, which may also lead to biased results. Second, due to the limitations of the included RCTs, the ERAS protocol employed across all the included RCTs was not similar. This discrepancy may potentially lead to biased results. Third, since ERAS in patients undergoing radical cystectomy has been largely reported in recent years, our study is limited by a lack of novelty. Lastly, a meta-analysis of randomized trials has its limitation such as publication bias. To ratify our findings, more high-quality RCTs with suitable study cohorts are needed to ascertain the efficacy and safety of ERAS and CRAS on perioperative outcomes of radical cystectomy. Regardless of these limitations, we believe that the ERAS protocols provide a theoretical basis for caring for patients undergoing radical cystectomy.

## Conclusions

Compared with CRAS protocols, the ERAS protocols on perioperative outcomes of radical cystectomy provide a better improvement of the length of hospital stay, time to first flatus, and time to regular diet. Additionally, ERAS protocols did not increase the risk of adverse events, when compared with CRAS protocols.

## Data Availability Statement

The datasets analyzed during the current study are available from the corresponding author on reasonable request.

## Author Contributions

JWu and YC designed the research, interpreted the data, and revised the paper. DZ, KS, TW, GW, and JWa performed the data extraction and carried out the meta-analysis. DZ drafted the paper. All of the authors approved the submitted and final versions.

## Conflict of Interest

The authors declare that the research was conducted in the absence of any commercial or financial relationships that could be construed as a potential conflict of interest.
